# Anomalous systemic arterial supply to the left lower lung lobe: A case report

**DOI:** 10.3389/fmed.2022.904431

**Published:** 2022-07-22

**Authors:** Zhuo Wu, Baoning Xu, Di Zhou, Xueying Yang

**Affiliations:** Department of Thoracic Surgery, The Fourth Affiliated Hospital of China Medical University, Shenyang, China

**Keywords:** anomalous systemic arterial supply, coil embolization, pulmonary infarction, case report, lung segment

## Abstract

**Background:**

An anomalous systemic arterial supply to the lung lobes is a rare congenital pulmonary vascular malformation. Current treatments include thoracoscopic lobectomy, anatomical segmentectomy, simple ligation and arterial embolization. However, the optimal treatment remains controversial.

**Case presentation:**

A 29-year-old man was diagnosed with anomalous systemic arterial supply to the left lower lobe through contrast-enhanced computed tomography and three-dimensional reconstruction. He underwent coil embolization of the anomalous artery and was followed up for 1 year.

**Conclusions:**

Blockage of the blood flow of the anomalous systemic artery alone does not improve the blood supply of the pulmonary artery to lung tissue and thus cannot restore normal gas exchange through the blood-gas barrier. Coil embolization of the anomalous arterial supply can cause early postoperative pulmonary infarction.

## Introduction

An anomalous systemic arterial supply to the lung lobes is a rare congenital pulmonary vascular malformation, with that occurring to the basal segments of the left lower lobe being most common. In 1946, Pryce defined this condition as intralobar pulmonary sequestration but later realized that this disorder was significantly different from pulmonary sequestration (1, 2). Although the affected pulmonary segment is supplied by anomalous arteries, there is no lung tissue sequestration, and bronchial tree development is normal. Current treatment methods include surgery and interventional embolization. However, which method is better remains a matter of debate, and complication prevention and treatment require further observation and study. We share our experience by reporting the case of a patient who underwent embolization treatment at our hospital and presenting the results of a literature review.

## Case report

**Figure 1:**
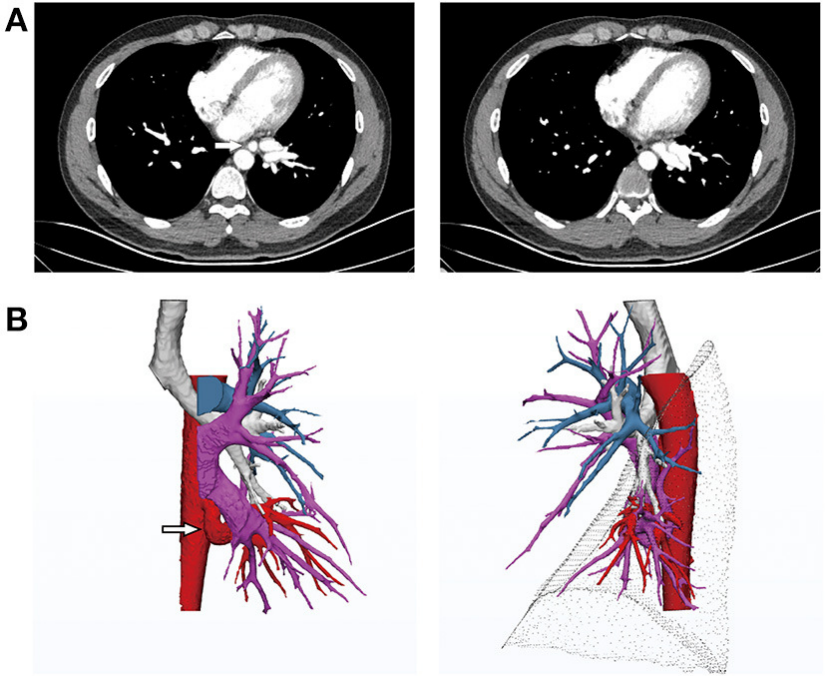
**(A)** Computed tomography angiography (CTA) before treatment revealing the anomalous systemic artery. **(B)** 3D reconstruction indicating normal development of the left bronchus. The anomalous systemic artery originated from the descending thoracic aorta and branched to the basal segments of the left lower lobe, parallel to the left inferior pulmonary vein. The pulmonary artery of the left lower lobe was absent from the basal segments and only present in the superior segment.

A 29-year-old man with intermittent hemoptysis (10 ml/episode) after physical activity sought treatment 1 year ago at our hospital. He became tired easily after regular physical activity. Computed tomography angiography (CTA) and three-dimensional computed tomography (3DCT) reconstruction showed a robust artery arising from the descending aorta, which was adjacent to the inferior pulmonary vein and approximately 2 cm in diameter. Branches of the artery intertwined with the inferior pulmonary vein in the basal segments. The superior segmental artery was present, whereas the basilar arterial trunk was absent. The structure of the bronchus in the left lower lobe was normal, and no lung tissue sequestration was noted (Figure 1). After consulting with the radiologist, it was determined that the superior segment was supplied by the pulmonary artery and that the basal segments were supplied by an abnormal systemic artery. The overall development of the patient was normal, and no obvious murmur was detected *via* auscultation of the heart and lungs. Ultrasonography revealed no abnormalities in the structure or function of the heart, and pulmonary function was normal.

**Figure 2:**
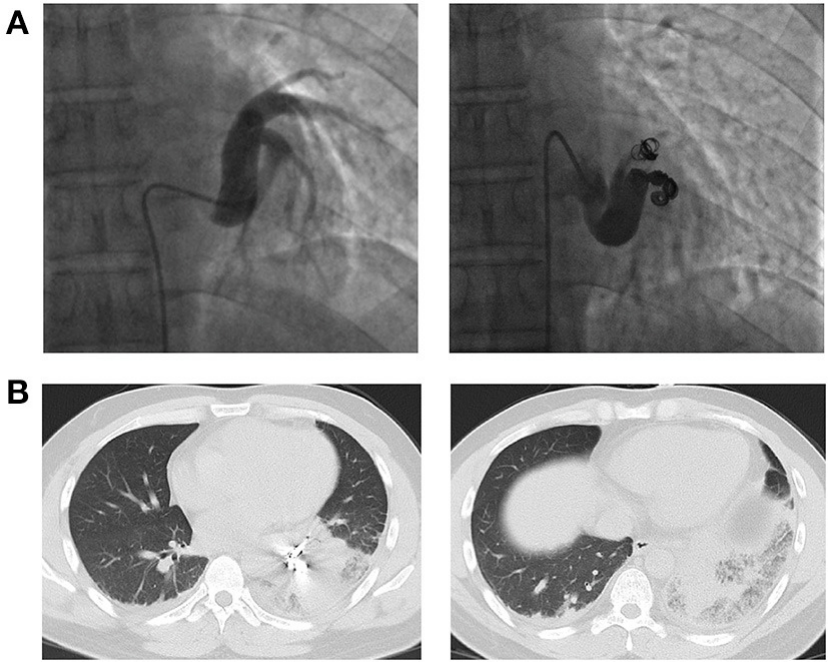
**(A)** Angiography and coil embolization for the anomalous systemic artery. **(B)** CT images showing pulmonary infarction at 48 h after the intervention.

After multidisciplinary treatment (MDT) by thoracic surgery, vascular surgery and interventional radiology, we decided to perform interventional therapy *via* coil embolization to preserve as much lung tissue as possible and protect pulmonary function and obtained the patient's consent. The procedure was uneventful (Figure 2A). However, after 24 h, the patient developed severe chest pain accompanied by labored breathing (a clinical manifestation of pulmonary infarction), and levels of serum fibrinogen and D-dimer were elevated. Forty-eight hours after the intervention, chest CT showed signs of infarction of the left lower lobe (Figure 2B). Serious symptoms of pulmonary infarction were observed at 72–96 h after the intervention and were accompanied by fever. Morphine was given for analgesia and sedation. His symptoms gradually resolved at 1 week after the intervention, and the patient was discharged at 2 weeks after the intervention.

**Figure 3:**
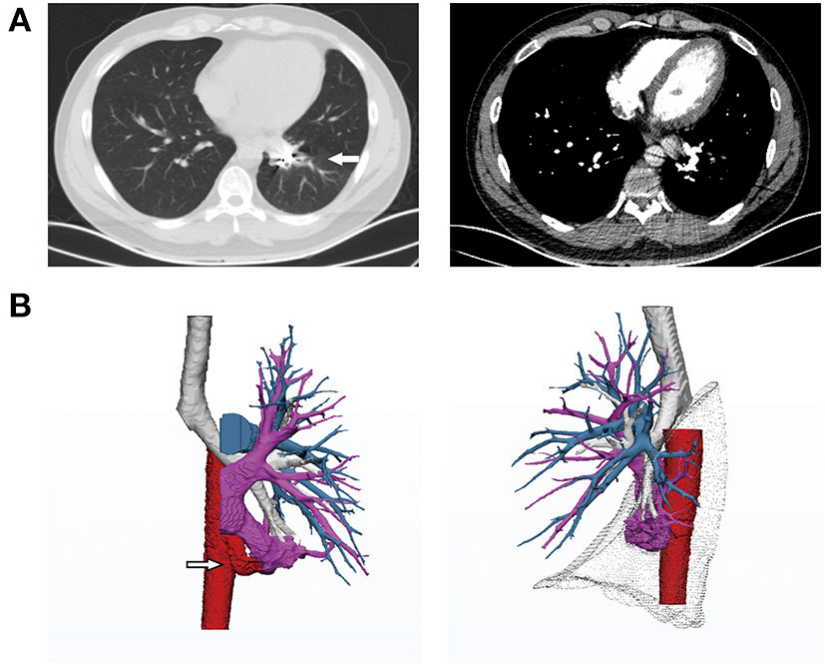
CT images at 1-year follow-up. **(A)** Lung CT showing significant alleviation of the interstitial changes in the left lower lobe. **(B)** 3D reconstruction showing blockage of the anomalous systemic artery and atrophy of the accompanying pulmonary vein in the basal segments but no change in the superior segment. The images of the pulmonary arteries were similar to those taken before the intervention. The pulmonary artery was absent from the basal segments.

At the 3-month follow-up, his hemoptysis had subsided completely, and his fatigue after physical activity had improved significantly. After 1 year of embolization, CTA and 3D reconstruction of the pulmonary arteries revealed that the anomalous systemic artery was completely blocked by the coils; the distal arterial branch was atrophic, and the accompanying inferior pulmonary venous branch showed no blood return. The inferior left pulmonary artery and the superior segmental vein remained unchanged (Figure 3). No coils were found to be coughed up during follow-up.

## Discussion

Controversy exists regarding whether an anomalous systemic arterial supply to normal lungs is different from typical pulmonary sequestration. According to Pryce's classification, an anomalous systemic blood supply to the basal segments is considered a type of pulmonary sequestration (1). However, a normal bronchial tree is present in the abnormal lung tissue and there is no sequestration of the pulmonary parenchyma with an anomalous systemic arterial supply, unlike what occurs with pulmonary sequestration. Moreover, the pulmonary artery is absent from the lung segment in cases of anomalous arterial supply (2–4). As a result of the lack of consensus, several similar terms have been proposed, including “arterial pulmonary malinosculation” and “systemic arterialization of the lung without sequestration”, but the most widely accepted term is “anomalous systemic arterial supply” (5, 6).

Although the cause of this disorder remains unclear, most researchers agree that it may be due to plexiform lesions of the primitive pulmonary arterial branches in the embryonic stage, which cause the primitive branches from the aorta to supply the lung buds instead of degenerating. Branches of the pulmonary artery from the lower lobe, i.e., the basal segments, are usually absent (7, 8). Different degrees of defects may lead to different degrees of variation.

Cough, expectoration and recurrent pneumonia are the most common symptoms of patients with pulmonary sequestration (9). Unlike with pulmonary sequestration, the main symptom of an anomalous systemic arterial supply to the basal segments of the left lower lobe is hemoptysis (10–12). This is because most of the anomalous systemic arteries originate from the descending aorta and are usually large in diameter, which can cause increased pressure in the pulmonary capillaries and pulmonary veins. Pulmonary capillaries and veins under excessive pressure are prone to rupture, causing intra-alveolar hemorrhage (13). Persistent pulmonary venous hypertension can lead to dilation of the left atrium, congestive heart failure, coughing, wheezing after physical activity, chest pain and even breathing difficulty, especially in people with a poor physical condition.

The current standard treatment for pulmonary sequestration is surgical resection. For symptomatic patients with extralobar or intralobar sequestration, surgical options include lobectomy, wedge resection, and anatomical segment resection (14, 15). There is controversy regarding treatment of an anomalous systemic arterial supply to the basal segments of the left lower lobe. Treatment has progressed from initial lobectomy by thoracotomy to thoracoscopic lobectomy, anatomical segment resection (16), and thoracoscopic anastomosis between anomalous systemic arteries and pulmonary arteries. However, due to the long-term high-pressure blood flow in the anomalous systemic arterial supply in adults, the blood vessel wall thickens and becomes less elastic, which results in poor blood perfusion after anastomosis. Some researchers suggest that vascular anastomosis is more suitable for children, who have been affected by this disorder for a shorter time period than adults (17, 18). Because anomalous systemic arteries provide blood to normal lung lobes with ventilation, surgeons began to attempt simple thoracoscopic ligation of anomalous systemic arteries or arterial embolization to preserve more lung tissue and reduce trauma (19–21).

The main symptom of the patient reported in this study was hemoptysis. Preoperative contrast-enhanced CT and 3D reconstruction of the pulmonary arteries showed that the patient's anomalous systemic artery originated from the descending thoracic aorta and supplied the basal segments of the left lower lobe. No obvious abnormalities in the bronchial tree were observed, though the pulmonary artery was absent from the basal segments. The pulmonary artery developed normally in the superior segment, and only one vein in the superior segment drained to the inferior pulmonary vein. Based on comprehensive analysis of the patient's medical history and imaging findings, we performed embolization of the anomalous systemic artery. The patient developed severe pulmonary infarction after the intervention; with rescue treatments, his condition gradually stabilized at 1 week. CTA and 3D reconstruction of the pulmonary arteries at 1 year after the intervention showed complete occlusion of the anomalous artery and atrophy of the accompanying inferior pulmonary vein in the basal segments. No compensatory growth of the pulmonary arteries was observed, and the basal segments of the left lower lobe were partially collapsed, with interstitial exudation.

The goal of treating hemoptysis through embolization was achieved in this patient. However, the pulmonary infarction that occurred during treatment increased his risk and caused the patient to develop scar tissue in response to the treatment, and the basal segments of the left lower lobe were left functionless. Through this case study, it can be concluded that blockage of the blood flow of an anomalous systemic artery alone does not improve the blood supply of the pulmonary artery to the lung tissue and thus cannot restore normal gas exchange through the blood-gas barrier. To confirm whether a reserved lung segment benefits patients, a study with a large sample size and long-term follow-up is needed. Overall, application of contrast-enhanced CT and 3D reconstruction of the pulmonary arteries can aid in the diagnosis and treatment of this disorder.

## Data availability statement

The original contributions presented in the study are included in the article/supplementary material, further inquiries can be directed to the corresponding author/s.

## Ethics statement

The studies involving human participants were reviewed and approved by Ethics Committee of the Fourth Hospital of China Medical University (EC-2020-KS-043). The patients/participants provided their written informed consent to participate in this study. Written informed consent was obtained from the individual(s) for the publication of any potentially identifiable images or data included in this article.

## Author contributions

ZW prepared the initial manuscript. XY edited and submitted the manuscript. ZW and XY drafted the article and gave final approval of the version to be published. BX and DZ were involved in the diagnosis and treatment of the patient. All authors have read and approved the final manuscript.

## Funding

This work was supported by Scientific Research Projects of Education Department of Liaoning Province (#ZF2019024).

## Conflict of interest

The authors declare that the research was conducted in the absence of any commercial or financial relationships that could be construed as a potential conflict of interest.

## Publisher's note

All claims expressed in this article are solely those of the authors and do not necessarily represent those of their affiliated organizations, or those of the publisher, the editors and the reviewers. Any product that may be evaluated in this article, or claim that may be made by its manufacturer, is not guaranteed or endorsed by the publisher.
